# Evolution of the Inhibitory and Non-Inhibitory ε, ζ, and IF_1_ Subunits of the F_1_F_O_-ATPase as Related to the Endosymbiotic Origin of Mitochondria

**DOI:** 10.3390/microorganisms10071372

**Published:** 2022-07-07

**Authors:** Francisco Mendoza-Hoffmann, Mariel Zarco-Zavala, Raquel Ortega, Heliodoro Celis-Sandoval, Alfredo Torres-Larios, José J. García-Trejo

**Affiliations:** 1 Facultad de Ciencias Químicas e Ingeniería, Universidad Autónoma de Baja California (UABC)—Campus Tijuana, Tijuana C.P. 22390, Baja California, Mexico; 2 Departamento de Biología, Facultad de Química, Ciudad Universitaria, Universidad Nacional Autónoma de México (U.N.A.M.), Ciudad de Mexico C.P. 04510, Coyoacan, Mexico; 3 Instituto de Fisiología Celular (IFC), Ciudad Universitaria, Universidad Nacional Autónoma de México (U.N.A.M.), Ciudad de Mexico C.P. 04510, Coyoacan, Mexico

**Keywords:** ATP, synthase, zeta, ζ, epsilon, ε, IF_1_, evolution, mitochondrial, endosymbiosis

## Abstract

The F_1_F_O_-ATP synthase nanomotor synthesizes >90% of the cellular ATP of almost all living beings by rotating in the “forward” direction, but it can also consume the same ATP pools by rotating in “reverse.” To prevent futile F_1_F_O_-ATPase activity, several different inhibitory proteins or domains in bacteria (ε and ζ subunits), mitochondria (IF_1_), and chloroplasts (ε and γ disulfide) emerged to block the F_1_F_O_-ATPase activity selectively. In this study, we analyze how these F_1_F_O_-ATPase inhibitory proteins have evolved. The phylogeny of the α-proteobacterial ε showed that it diverged in its C-terminal side, thus losing both the inhibitory function and the ATP-binding/sensor motif that controls this inhibition. The losses of inhibitory function and the ATP-binding site correlate with an evolutionary divergence of non-inhibitory α-proteobacterial ε and mitochondrial δ subunits from inhibitory bacterial and chloroplastidic ε subunits. Here, we confirm the lack of inhibitory function of wild-type and C-terminal truncated ε subunits of *P. denitrificans*. Taken together, the data show that ζ evolved to replace ε as the primary inhibitor of the F_1_F_O_-ATPase of free-living α-proteobacteria. However, the ζ inhibitory function was also partially lost in some symbiotic α-proteobacteria and totally lost in some strictly parasitic α-proteobacteria such as the Rickettsiales order. Finally, we found that ζ and IF_1_ likely evolved independently via convergent evolution before and after the endosymbiotic origin mitochondria, respectively. This led us to propose the ε and ζ subunits as tracer genes of the pre-endosymbiont that evolved into the actual mitochondria.

## 1. Introduction

**Figure 1:**
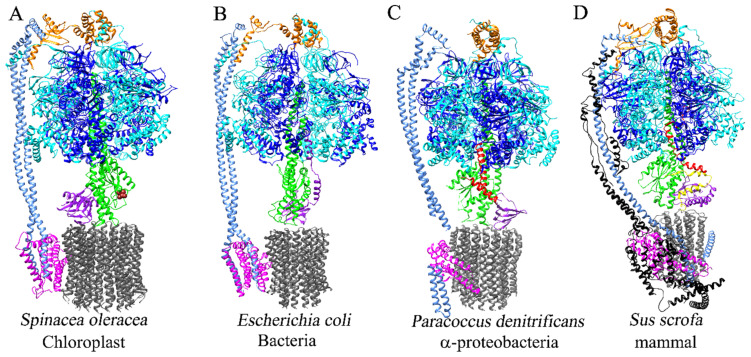
**Structures of the F_1_F_O_-ATP synthases from chloroplast, bacteria, α-proteobacteria, and mammals.** (**A**) The chloroplast enzyme is represented by the F_1_F_O_-ATP synthase of *Spinacea oleracea* (PDB_id 6VON). The regulatory cysteines in the γ subunit are shown in brown spheres. (**B**) The bacterial F_1_F_O_-ATP synthase is represented by *E. coli* (PDB_id 5T4O), which is inhibited by its ε subunit (purple). (**C**) The α-proteobacterial F_1_F_O_ is represented by *P. denitrificans* (PDB_id 5DN6), which is inhibited by its ζ subunit (red). (**D**) The mitochondrial F_1_F_O_-ATP synthase is represented by *Sus scrofa* (PDB_id 6J5I), which is inhibited by its IF_1_ subunit (red). Color coding of the subunits is as follows: in (**A**–**D**): α cyan; β blue; γ green; d or OSCP (for mitochondria) orange; ε or δ (for mitochondria) purple; mitochondrial ε in yellow; IF_1_ and ζ subunits in red; subunit *a* in magenta (Sub. 6 for mitochondria, and sub IV for chloroplast); subunit *b*, *b’* in pale blue (subunit I and II for chloroplast); subunits *c* in dark gray (subunit III for chloroplast); all supernumerary subunits of mitochondria in black (A6L, d, e, f, g, i/j, F6, etc.). The images were constructed using UCSF Chimera.

The F_1_F_O_-ATP synthase/ATPase is a reversible nanomotor. It can synthesize or hydrolyze MgATP according to the kinetic and thermodynamic conditions in the energy-transducing membranes of bacteria, mitochondria, and chloroplasts [1,2,3]. When the transmembrane proton gradient decreases, the F_1_F_O_ nanomotor is prone to rotate counterclockwise (CCW, viewed from F_O_ to F_1_) and work as a proton-pumping ATPase [4,5]. This ATPase activity thus threatens the cellular bioenergetics by decreasing the intracellular ATP pools. Given that this “reverse” F_1_F_O_-ATPase activity may exert a futile consumption of the intracellular ATP, the enzyme evolved in such a fashion that meant it acquired several control mechanisms that prevent this risky F_1_F_O_-ATPase activity. These inhibitory mechanisms are as follows: (1) Most if not all F-ATP synthases have an intrinsic kinetic MgADP inhibitory mechanism that exerts partial, i.e., not total inhibition [6]; (2) a bacterial ε subunit that exerts partial inhibition in several bacteria [7], with the exception of α-proteobacteria, where it is non-inhibitory, and the homologous ε subunit of the chloroplast F-ATP synthase (CF_1_F_O_) preserved its function as a partial inhibitor [8]. This is likely derived from cyanobacteria which also preserves ε inhibition [9,10], as embraced by the endosymbiotic theory [11,12,13]. (3) The ζ subunit of *Paracoccus denitrificans* and related α-proteobacteria exerts the total inhibition of the PdF_1_F_O_-ATPase activity [14]. (4) A mitochondrial inhibitor protein or IF_1_ which blocks completely and preferably the mitochondrial F_1_F_O_-ATPase activity [15]. (5) A redox and light-regulated disulfide bridge exclusive of the γ subunit of the chloroplastic F-ATP synthase (CF_1_F_O_) sensitive to dithiothreitol (DTT) [16,17], additional to the chloroplast inhibitory ε subunit (see Figure 1). (6) Another inhibitory protein named AtpΘ has been recently found in photosynthetic cyanobacteria; however, it is only a partial F_O_ inhibitor of the cyanobacterial ATP synthase [18,19]. (7) A different inhibitory mechanism of latent ATP hydrolysis has also been described in mycobacteria, in which an extra C-terminus of the α subunit of about 3.5 kDa interacts with subunit γ [20,21] and leads to a novel anti-tuberculosis compound [22,23]. In summary, nature evolutively designed several F_1_F_O_-ATPase inhibitory proteins or domains to complement the insufficient kinetic and partial inhibitory MgADP regulation. The evolutionary pressure of preserving ATP pools through nature makes it very evident, if not obvious, that partial kinetic MgADP inhibition is not enough to completely prevent futile F_1_F_O_-ATPase activity in all energy-transducing membranes. We briefly discuss each particular control case of the F-ATP synthases from non-α-proteobacteria, α-proteobacteria, and mitochondria and the critical implications for the endosymbiotic origin of the latter. First, we briefly describe our Materials and Methods for this evolutionary review analysis, and later, the natural inhibitory proteins of the F_1_F_O_-ATPase s in the Results and Discussion. 

## 2. Materials and Methods

### Construction of the Phylogenetic Trees of the ε and ζ Subunits

The phylogenies and cladograms of the ε and ζ subunits were carried out in a similar way using the maximum likelihood method, as described below for the ζ subunit. In the case of the ε subunits, a total of 137 protein sequences of F-ATP synthases’ ε subunits were aligned. The latter ε sequences were derived from α-proteobacteria, other bacteria, chloroplastidic ε subunits, and homologous mitochondrial δ subunits. With all these ε sequences, a maximum likelihood phylogenetic tree was constructed as described below. The latter procedure provided the best results according to the bootstrap analysis. To assess the ancestry/distribution of the ζ subunit in the α-proteobacteria class, the orders, families, and genera were identified following the taxonomy from the Patric database (https://www.patricbrc.org, last accessed on 1 June 2022). To have a representative sample of the whole class of the α-proteobacteria, we downloaded sequences from species belonging to representative genera and families of each order. As a result, we used representative sequences from each genus of the families. However, certain families had too many genera (from Rhodobacterales: Rhodobacteraceae, 118 genera, Rhodospirillales: Acetobacteraceae, 45 genera, and from Rhodospirillaceae, 28 genera). A representative number of sequences was then selected depending on the number of genera of each family. In total, 136 sequences were used. The sequences were retrieved from NCBI protein database. A delta blast (NCBI) was performed to obtain each sequence, where the ζ subunit from *P. denitrificans* was used as the reference sequence, and the bacteria of interest were selected to make the blast in each specific genome. The aminoacid (a.a.) sequence was compared with the reference to verify we had a ζ sequence. This process was repeated with each organism selected for this phylogeny. From the 136 sequences used, 36 were from Rhodobacterales, 59 from Rhizobiales, 14 from Rhodospirilalles, 10 from Sphingomonadales, 6 from Caulobacterales, 4 from Holosporales, 2 from Sneathinellales, 2 from Parvularculales, 1 from Pelagibacterales, 1 from Kiloniellales, and 1 from Kordimonadales. The sequences were then aligned using MAFFT [24] with default parameters, followed by the maximum likelihood [25,26] phylogeny carried out using the aligned sequences with PhyML [27], and the substitution model LG and 100 bootstraps were used, leaving the remaining settings as the defaults. The resulting phylogeny was visualized using iTOL (Interactive Tree Of Life (iTOL) v5 [28]. It is worth mentioning that the ζ sequences were updated since some previously had internal, N-terminal, or C-terminal gaps [29]; we removed those gaps in the actual phylogenetic analysis.

## 3. Results and Discussion

### 3.1. Inhibitory and Non-Inhibitory ε Subunits of the Bacterial F_1_F_O_-ATPases

Three inhibitory subunits regulate the bacterial F_1_F_O_-ATPase hydrolytic activity: the ε subunit, the ζ subunit, and the recently discovered AtpΘ subunit. The ε subunits works as an inhibitor, besides its central rotor structural role, in several bacteria and chloroplasts. [7]. Additionally, relatively recently, we discovered a newly inhibitory “zeta” (ζ) subunit of the F_1_F_O_-ATPase in *Paracoccus denitrificans*, a bacterium that belongs to the class of the α-proteobacteria from the phylum proteobacteria [14,30]. The cyanobacterial AtpΘ inhibitor subunit has been described very recently, but because there is no detailed structural and functional characterization of AtpΘ, it is not discussed here [18,19]. The primary function of ε is not the inhibition of the F_1_F_O_-ATPase; it is rather an essential structural subunit that connects the F_1_ and F_O_ moieties of the enzyme by contacting the central rotary γ subunit with the c-subunit ring of F_O_. This is evidenced by the fact that the genetic removal of the ε subunit in *Escherichia coli* produces structural uncoupling via physical separation of the F_1_ and F_O_ sectors and the concomitant accelerated soluble F_1_-ATPase activity. The soluble F_1_-ATPase activity in the bacterial cytoplasm produces a severe energy deficit that slows down bacterial growth [31]. Thus, ε is not always a strict inhibitor but a structural subunit and essential component of the central rotor of the F_1_F_O_-ATP synthase rotor, which is formed by the γ/ε/c_8-15_ subunits [32,33,34]. In some bacteria, ε acquired an inhibitory role, and this was firstly found via the purification of the *E. coli* F_1_-ATPase (EcF_1_-ATPase), where the enzyme became increasingly activated as soluble F_1_-ATPase due to the progressive dissociation of the *E. coli* ε subunit [7]. Furthermore, the reconstitution of the purified ε partially decreased the EcF_1_-ATPase, i.e., the inhibition by ε did not reach full inhibition [7]. The ε subunit has been extensively studied. In addition to resolving its C-terminal 2-helix bundle and N-terminal β-sheet barrel in solution [35], it was also found that the inhibitory domain resides in its two C-terminal α-helixes. This C-terminal domain undergoes large conformational changes due to the extension of this α-helical C-terminus reaching both the C-termini of α and β subunits of F_1_ and the central rotary γ subunit [33]. These first structural analyses later led to the finding that the extended conformer of ε blocks the gyration of the central rotor (Figure 1A, brown). In contrast, the compact ε conformer with the two C-terminal α-helixes forming a folded hairpin does not inhibit the EcF_1_-ATPase (the compact conformer is shown by the homologous mitochondrial δ subunit, Figure 1C, brown). Previously, it was also found that the extended conformer of ε works like a ratchet by inhibiting the counterclockwise (CCW) rotation of γ (viewed from F_O_ to F_1_). This was the first time that a ratchet inhibitory mechanism was proposed to explain the partial inhibition of ε on the *E. coli* F_1_F_O_-ATPase (EcF_1_F_O_) [36].

Recently, this compact/extended (or also called “up” and “down”) conformational change in ε has been structurally described in detail [37] and also detected with new approaches of synthetic biology and cross-linking in vivo, besides high-throughput electrophoresis and Western blot [38].

The conclusion is that this ratchet inhibitory mechanism selectively blocking the F_1_F_O_-ATPase activity occurs in vitro as well as in vivo, which has been recently called a gear-shifting mechanism, with some proposed intermediate states resembling the mechanical shift controlling the changes in velocity in automobiles [38]. However, some of us have recalled that these conformational changes in ε have been described previously via cross-linking in vitro and via crystallography and cryo-electron microscopy more recently [37], without a description of intermediate stages in the compact/extended conformation [39]. Therefore, we propose that the original ε ratchet mechanism [36] or “all or none” rotary movement is controlled by the compact or extended conformation of ε. Perhaps the closest to an intermediate conformation in this “up” or “down” transition is the first structure of the isolated structure of the γ/ε complex of the *E. coli* ε subunit resolved via crystallography [40]. Therefore, a pawl/ratchet mechanism, as we also proposed for the inhibitory subunit ζ in *Paracocus denitrificans* [41,42,43,44] (see below), seems more likely to take place as the originally proposed ε ratchet mechanism than a gear/shift one.

Another interesting feature of the bacterial ε subunit is that in some bacteria such as *E. coli*, and more significantly in *Thermophilic PS3* bacterium, these ε subunits have a regulatory MgATP-binding site, which is additional to the six nucleotide-binding sites of the F_1_-ATPase [45,46]. This ATP-binding site is of variable affinity in different bacterial species, for instance, the *E. coli* ε subunit has a low ATP-binding affinity in the mM range (22 mM) [47], whereas in the *Thermophilic bacterium PS3* ε subunit, the MgATP-binding affinity is higher since the Kd is in the μM range (1.4 μM) at 25 °C; however, this Kd increases to a more physiological mM range of ATP concentrations at higher physiological temperatures for thermophilic bacteria [48]. This MgATP-binding site is highly specific for MgATP since it also binds MgADP but with much lower affinity (130 μM), i.e., the affinity of PS3-ε for MgATP was about 100-fold higher for MgATP than for MgADP [48]. In general, it is considered that these inhibitory bacterial ε subunits bind ATP rather than ADP, and that this ATP-binding mechanism works as a cellular ATP sensor. Binding MgATP stabilizes the ε compact or non-inhibitory conformation and thus promotes F_1_F_O_-ATPase activity, consuming the cytosolic ATP when it exists in excess. On the other hand, the dissociation of MgATP from the ε subunit of these bacterial F-ATP synthases stabilizes the extended and inhibitory ε conformation, thus selectively blocking the F_1_F_O_-ATPase activity and therefore preventing the futile consumption of ATP when it is scarce in the bacterial cytoplasm. This ATP binding to ε occurs in an ATP-binding motif found conserved in these inhibitory ε subunits, which has the consensus sequence I(L)DXXRA. This ATP-binding motif is located mainly on the two α-helix hairpins of the C-terminal inhibitory domain, having few contacts with the N-terminus β-barrel of these ε subunits [47]. When ATP is bound to ε at this binding site, it stabilizes the compact non-inhibitory ε conformer, explaining the ATP sensor role of these ε subunits. 

**Figure 2:**
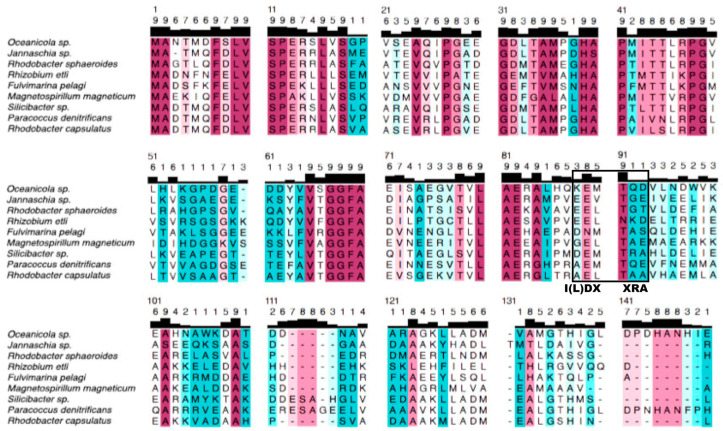
**Alignment of the ε subunits of the α-protebacterial ATP synthase.** The sequences shown were aligned with Clustal Omega, and the alignment was visualized in UCSF Chimera. Color codes: cherry red: strongly conserved residues; pink, conserved residues; light pink, less conserved residues; 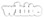, slightly variable residues; light blue, variable residues; pale blue, average variable residues; strong blue, strongly variable residues. The most conserved region of the Pd-ε subunit is in the N-terminal side, the function of which is structurally connecting the γ subunit of F_1_ with the c_12_ ring of F_O_; see the case of the *P. denitrificans* F-ATP synthase in Figure 1C. The Pd-ε subunit is the largest one of this alignment, even larger than that of *Oceanicola* sp. with additional 9 a.a.s at the C-terminus. The C-terminus of α-proteobacterial ε subunit is highly variable, and this divergence leads to the loss of the ATP-binding motif of other bacterial inhibitory ε subunits (I(L)DXXRA) (see black box). The divergence of the C-termini of the α-proteobacterial ε subunits very likely produces the loss of both the inhibitory function of the two C-terminal α-helical hairpin domains and the loss of the regulatory ATP-sensor-binding motif. See text for further details.

**Figure 3:**
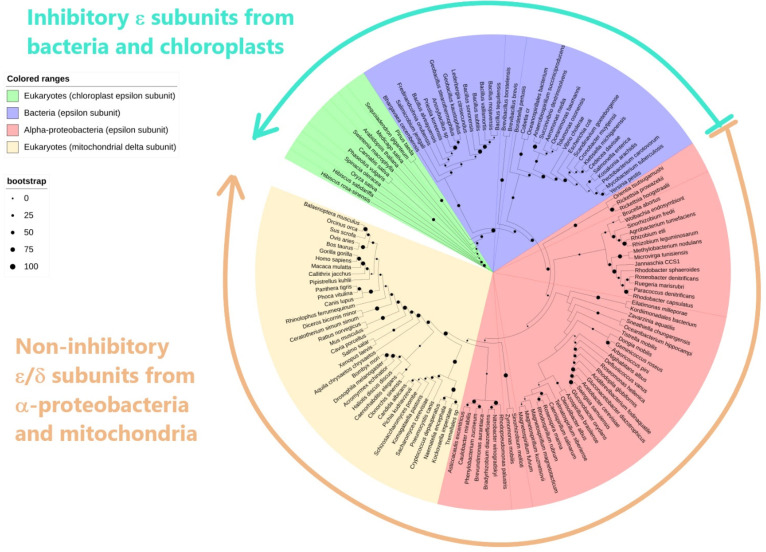
**Cladogram of the ε subunit of the α-proteobacterial F-ATP synthase.** A total of 137 protein sequences of ε subunits from α-proteobacteria (red), other bacteria (blue), chloroplast ε subunit (green), and homologous mitochondrial δ subunits (yellow) were aligned, and a maximum likelihood phylogenetic tree was constructed as detailed in the text. Green arrow: inhibitory ε subunits of bacteria and chloroplasts. Orange arrow: non-inhibitory ε and δ subunits of α-proteobacteria and mitochondria, respectively.

**Figure 4:**
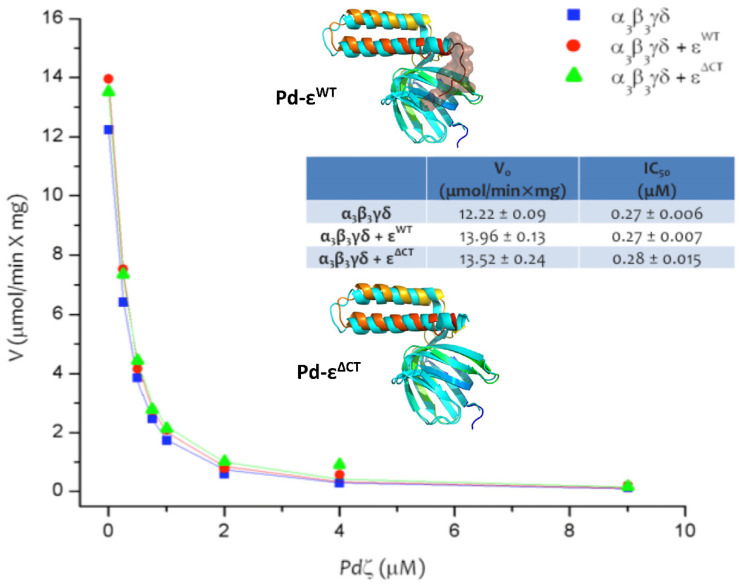
**Non-inhibitory wild-type and C-terminal truncated ε subunits of the F-ATP synthase of *Paracoccus denitrificans*.** In total, 10 μg of purified α_3_β_3_γδ complex (lacking both ε and ζ) was reconstituted by 30 min with the indicated amounts of ζ. Afterwards, the hydrolytic activity was started by adding 10 mM of MgATP, incubating for 1 min at 37 °C and arresting the reaction with 6% of TCA. The specific initial PdF_1_-ATPase activity was calculated via the colorimetric determination of the released P_i_ (see Refs. [14,29] and Methods). Y axis: specific PdF_1_-ATPase activity (V = μmol of ATP hydrolyzed/(min. × mg. of protein). X axis: concentration of the recombinant wild-type ζ subunit from *P. denitrificans* [Pdζ] in μM scale. (The blue squares (■) show the reconstitution of the Pd-ζ subunit on the α_3_β_3_γδ complex, while in red circles and green triangles (●,▲) an excess of the wild-type (Pd-ε^WT^) or the C-terminal truncated (Pd-ε^ΔCT^) subunits were previously reconstituted, respectively, to the α_3_β_3_γδ complex by 30 min before the ζ reconstitution (see text and Refs. [14,29] for further details)). Insets: structure of the Pd-ε^WT^ (blue to chocolate rainbow, N- to C-terminus) and Pd-ε^ΔCT^ subunits (blue to red rainbow, N- to C-terminus); structural models are superimposed to the wild-type ε subunit from *E. coli*’s F-ATP synthase (PDB_id 1AQT, cyan). The truncated 9 a.a. in Pd-ε^ΔCT^ are shown in chocolate surface.

We previously assessed whether ε could or could not be inhibitory in *P. denitrificans* as a model bacterium of the α-proteobacteria class. In order to evaluate the putative inhibitory role of ε in *P denitrificans*, we cloned, over-expressed, and reconstituted a wild-type ε subunit of the F-ATP synthase of *P. denitrificans* into the F_1_F_O_ and F_1_ complexes or *P. denitrificans* (PdF_1_F_O_ and PdF_1_, respectively). The PdF_1_F_O_ used remained natively attached to inside-out membranes or Sub-Bacterial Particles (SBPs), whereas the PdF_1_-ATPase was isolated in a soluble form. In both cases, the ε subunit could not exert any inhibitory effect whatsoever in the PdF_1_F_O_-ATPase [14] or the PdF_1_-ATPase activities [29]. Furthermore, this inhibitory effect was observed in the newly discovered 11 kDa subunit which we named “zeta” (ζ). We named it this because of its molecular weight and migration in SDS-PAGE gels, which followed ε with a smaller size, and the following Greek symbol after ε is “ζ” [14]. The observation of a lack of inhibitory effect of the wild-type ε from *P. denitrificans* (Pd-ε) in the PdF_1_F_O_-ATPase may not be conclusive since the coupled F_1_F_O_-ATP synthase contains its endogenous ε subunit, otherwise F_1_ and F_O_ could detach from each other. Therefore, we conducted the reconstitution of excess wild-type Pd-ε into the soluble PdF_1_-ATPase, which lost a fraction of its content of endogenous ε and also in the Pdα_3_β_3_γδ complex prepared via immunoaffinity chromatography [29]. The latter preparation lacked both the endogenous ε and ζ subunits and was activated as ATPase. Thus, it was the best model, at that time, to assess conclusively whether ε has, or has not, any inhibitory function after its reconstitution in the F-ATPase of *P. denitrificans*. In both cases, the recombinant ε subunit was completely unable to exert any inhibitory activity or to modify the affinity with which ζ inhibited both enzymes [29]. Rather than inhibition, the recombinant Pd-ε exerted a slight but reproducible activation of the PdF_1_-ATPase and the Pdα_3_β_3_γδ complexes, attributable to the structural and functional stabilization of both complexes [29]. These results clearly showed that in *P. denitrificans*, and presumably in the rest of the α-proteobacteria, the inhibitory function was lost from α-proteobacterial ε and eventually acquired by the ζ subunit, according to the phylogeny and alignment analyses of this work (see Figure 1, Figure 2, Figure 3 and Figure 4). 

Recently, this putative inhibitory effect of Pd-ε was reconsidered by others [49] by constructing some C-terminal truncated Pd-ε mutants lacking parts of the putatively inhibitory domain of Pd-ε. The in vivo expression of these truncated constructs of Pd-ε did not exert a significant increase in the in vitro assays of PdF_1_F_O_-ATPase of PSB, as expected after removing a putatively “inhibitory” C-terminal two-α-helix hairpin of Pd-ε. Rather than the truncation of Pd-ε, it was the deletion of the endogenous ζ subunit, the one that exerted a 2-fold activation of the steady-state PdF_1_F_O_-ATPase of PSB [49]. Only when the PdF_1_F_O_-ATPase was activated under extreme conditions by non-physiological oxyanions such as selenite or the detergent lauryldimethylamineoxide (LDAO), it seemed that the truncated forms of Pd-ε exerted a slight decrease in the AC_50_ of these activators [49]. However, these experiments lack very important controls to show that, for instance, their coupled ATPase activity assay is not delivering inhibited or overestimated ATPase rates by carrying them out it in a multiwell device and in the presence of selenite, which may affect the enzymes of the coupled ATPase assay. In summary, some controls of all these parameters were not presented, and therefore, the apparent slight PdF_1_F_O_-ATPase activation with truncated Pd-ε subunits could result from kinetic artifacts rather than a true activation. In addition, selenite and LDAO are not physiological activators; thus, it does not seem plausible that these apparent inhibitory effects of Pd-ε could take place in vivo. Regardless of these details, their results confirmed that the removal of the Pd-ε C-terminus did not activate the PdF_1_F_O_-ATPase significantly, whereas the full deletion of ζ partially activated the PdF_1_F_O_-ATPase turnover [49]. Therefore, the inhibitory role of the α-proteobacterial F_1_F_O_-ATPase has shifted from ε to ζ.

To confirm whether the inhibitory and/or ATP-binding properties of inhibitory ε subunits from other bacteria are or are not preserved in the ε subunit of the F-ATP synthase of α-proteobacteria, we first analyzed the conservation of the ε subunit in the α-proteobacteria class, as we did before with the ζ subunit [29]. The α-proteobacterial ε alignments (Figure 2) showed high conservation in its N-terminus, similar to what we found for the ζ subunit [29]. However, in contrast to the ζ subunit, whose functional inhibitory domain is on its N-terminal side [29], the ε subunits have a non-inhibitory N-terminal β-barrel domain. The latter interacts with the c-ring of the central F-ATP synthase rotor and so it carries out the main structural function of ε (see Figure 1A, brown and cherry-red and pink segments in the alignment, Figure 2).

On the other hand, the most variable segment of α-proteobacterial ε was found all along the C-terminal hairpin of two α-helixes, corresponding to the inhibitory domain of bacterial inhibitory ε subunits. This clearly shows that the primary structural function of α-proteobacterial ε is well preserved in its β-barrel N-terminal domain. In contrast, the putatively inhibitory ε domain of the C-terminal hairpin of two α-helixes is largely variable (see white and blue colors in Figure 2). The fact that the N-terminal domain of ε is the main domain conserved in α-proteobacteria indicates that the primary F_1_-F_O_ connecting function of α-proteobacterial ε is the one that is preserved. In contrast, the C-terminal divergence shows that the inhibitory function was lost in most if not all cases in the α-proteobacterial class. In the majority of the α-proteobacterial ε subunits, the C-terminus is truncated (see Figure 2), which shows that in α-proteobacteria, this C-terminal two-α-helix bundle is dispensable. This C-terminus truncation is another indication that ε has lost its inhibitory properties in the α-proteobacterial class.

In addition, we also previously looked for the conservation of the ε MgATP-binding motif “I(L)DXXRA” of inhibitory ε subunits. According to structural alignments with the crystal structure of the PS3-ε:ATP complex (PDB_id 2E5Y), this motif should be in residues 86–93 of Pd-ε (see the black box in Figure 2). However, as can be seen in reference [50] and in Figure 2, in α-proteobacteria, this ATP-binding motif and other residues that interact with the ATP molecule are totally absent. This motif and the other ATP-interacting residues are located in the C-terminal side of other bacterial inhibitory ε subunits, so it is clear that ATP binding was no longer needed in these non-inhibitory α-proteobacterial ε subunits. Thus, the ATP sensor motif, together with the C-terminal, otherwise the inhibitory domain, were dispensable in α-proteobacteria.

Further analyses are discussed below to confirm that the ε subunit of α-proteobacterial F-ATP synthases only preserves its primary structural function and lacks the inhibitory one. Meanwhile, most available data reviewed here strongly suggest that is the case. Interestingly, when we first resolved the ζ’s NMR structure and inhibitory domain at the N-terminus of ζ in collaboration with Prof. Kurt Wüthrich [29,51], we also found and calorimetrically described a low-affinity nucleotide-binding site in the Pd-ζ subunit [29], which was confirmed by further NMR analyses [43]. This ζ nucleotide-binding site is reminiscent of the ATP-binding site of the bacterial inhibitory ε subunits [45,46,48] and likely plays an important role in the control of the inhibitory activity of ζ. However, the ATP-binding motif of inhibitory ε subunits is also absent in the ζ subunits of α-proteobacteria [29]. Therefore, a hitherto unknown nucleotide-binding motif must be present in ζ. This implies that not only the F-ATPase inhibitory function was transferred from ε to ζ in α-proteobacteria, but also the ATP binding or sensor capacity to control the F_1_F_O_-ATPase activity was acquired by the ζ subunit. 

To extend our structural/functional analyses, we compared the α-proteobacterial ε subunit with other bacterial inhibitory ε subunits such as that of *E. coli* and found a nine a.a. extension in the C-terminus of the Pd-ε subunit, which is absent in the α-proteobacteria analyzed (see Figure 2), and we confirmed that it is also absent in the inhibitory ε subunit of *E. coli* (see Figure 4). To complement the comparative analyses of the α-proteobacterial ε subunit, we compared it not only with other bacterial inhibitory ε subunits, but also with the homologous non-inhibitory δ subunit of the mitochondrial mtATP synthase. It has been shown that it is the mitochondrial δ and not the mitochondrial ε subunit which is homologous to the bacterial ε subunit. The mitochondrial ε (mt-ε) is a totally different and additional protein incorporated to the F-ATP synthase after the mitochondrial endosymbiosis. The mitochondrial δ subunit has a similar folding to the bacterial ε, but its orientation in the central rotor of the enzyme and its interaction with the mt-ε hinder any conformational changes in mitochondrial δ (compare Figure 1C,D, purple). Thus, mitochondrial δ (mtδ) and ε (mtε) subunits (purple and yellow in Figure 1D) only form a structural connection of the central rotor without any inhibitory role [52]. This seems to be the reason why a different mitochondrial inhibitor protein (IF_1_) emerged, to fully prevent the mitochondrial F_1_F_O_-ATPase activity mainly during anoxia or ischemia [15], since the mtδ and mtε subunits are unable to exert this inhibition.

In this context, and to extend the alignments to the phylogenetic analysis of the α-proteobacterial ε subunit of the F-ATP synthase, the sequence of the α-proteobacterial ε subunit was compared with the ε subunit from other bacteria, chloroplasts, and homologous mitochondrial δ subunits. We first carried out a comparative analysis between the bacterial ε sequences and the mt-δ, revealing that these are similar enough to cluster together and diverge from non-α-proteobacterial ε subunits (Figure 3). This ε-mtδ clustering seems to be associated with the loss of inhibitory function of the δ subunit of mitochondrial mtATP synthase. This also eventually promoted the evolutionary emergence of the inhibitory mitochondrial IF_1_, most likely in a post-mitochondrial endosymbiotic event. To show the phylogenetic relationships of the α-proteobacterial ε with its bacterial and mitochondrial homologs, we carried out a cladogram of the evolution of the α-proteobacterial ε subunits using the maximum likelihood method with 137 bacterial, α-proteobacterial, and mitochondrial mt-δ homologs (details of how this analysis was conducted are described in the Materials and Methods and in the section “Distribution of the ζ subunit among the α-proteobacterial class”). 

We found very strikingly that the resulting phylogenetic tree is clearly split into two “functional” clusters. One cluster (green arrow, Figure 3) embraces *inhibitory* ε subunits of other bacteria, out of the α-proteobacteria class (blue, Figure 3), such as those of the *E. coli* or the extremophile *Geobacillus stearothermophilus* (formerly known as *Bacillus PS3*); these also include the *inhibitory* ε subunit of chloroplasts. A second cluster (blue, Figure 2) contains those εs belonging to the *non-inhibitory* mitochondrial δ subunits and the *non-inhibitory* ε subunits from α-proteobacteria, such as the one from *Paracoccus denitrificans*.

Although the ε cladogram is clearly split into *inhibitory* (bacteria and chloroplasts) and *non-inhibitory* ε subunits (from α-proteobacteria and mitochondria), we did not discard the possibility that some exceptions to the general *non-inhibitory/inhibitory* trends may occur in α-proteobacterial ε subunits, and thus there may be examples in this class that will probably preserve some inhibitory function. On the other hand, we also considered that in the branches of *inhibitory* ε subunits, there may be some instances where this ε subunit is unable to exert inhibition. For instance, the ε subunit of the F-ATP synthase from *Bacillus subtilis* (Bs-ε) does not exert F_1_-ATPase inhibition; this Bs-ε even induces the significant activation of its respective bacterial F_1_-ATPase.

This seems to take place because the Bs-ε is somehow relieving the intrinsic kinetic MgADP inhibition [53]. This Bs-ε activating effect has also been demonstrated in the full BsF_1_F_O_-ATPase [54]. Furthermore, it has also been shown that the last five residues of the very C-terminus of the ε subunit of the F-ATP synthase from *E. coli* (Ec-ε) enhance the clockwise F-ATP synthase turnover and the respiratory *E. coli* growth. This effect is probably related to its ratchet or unidirectional inhibitory mechanism, and these inhibitory C-terminal residues are variable between Gram-positive and Gram-negative bacteria [55]. The latter divergence of the C-termini in bacterial F-ATP synthase ε subunits may induce the loss of the F_1_-ATPase inhibitory ε function, as is shown here in the ε subunit of the F-ATP synthase of α-proteobacteria. In summary, the bacterial ε subunit, besides being a partial and not total inhibitor, may occasionally be an activating subunit rather than an inhibitory one. This shows on the one hand that the “*inhibitory*” or “*non-inhibitory*” character or classification of the F-ATP synthase ε subunit shown in Figure 3 may have some exceptions. In addition, this also shows more clearly that the primary function of ε is not F_1_F_O_-ATPase inhibition but rather structural by connecting the *c*-ring of F_O_ with the central γ subunit to form the full rotor of this nanomotor with the γ/ε/c_8-15_ subunits [34]. It sometimes happens that in several bacteria, the ε subunits acquire an additional and partial inhibitory role, but this is not the case for all bacteria. There is a wide range of bacteria where the *inhibitory* or *non-inhibitory* character of ε is yet to be confirmed. Another instance of ε activation instead of inhibition is indeed the F_1_F_O_-ATPase of *P. denitrificans*, which we showed by several means that it did not exert any F_1_-ATPase inhibition at all of the isolated PdF_1_-ATPase after the removal of the endogenous ε (leaving the α_3_β_3_γδ complex) [29]. Besides, ε did not modify the inhibition exerted by the novel inhibitory ζ subunit [29], and furthermore, the reconstitution of Pd-ε produced a partial but consistent modest activation of the PdF_1_-ATPase [29] (see also Figure 4). We explain this partial activation by the structural and functional stabilization of the native PdF_1_-ATPase after reconstitution into an enzyme that has become destabilized by the partial or total release of its endogenous ε subunit during purification [29]. We will refer to this point below. 

As mentioned above, the C-terminus of Pd-ε is nine aminoacid residues (a.a.s) longer than its respective homologous in α-proteobacteria and also nine a.a.s longer than the inhibitory ε subunit of *E. coli* (see Ref. [50], and Figure 2). Therefore, to evaluate if this extra C-terminal segment could somehow hinder the Pd-ε inhibitory function by preventing the compact/extended conformational changes needed to exert F_1_-ATPase inhibition, we constructed a C-terminal truncated Pd-ε^ΔCT^ mutant lacking the last nine residues to determine if this construction could inhibit the PdF_1_-ATPase. To appraise this, we reconstituted both the wild-type Pd-ε and the Pd-ε^ΔCT^ mutant to the PdF_1_ and Pdα_3_β_3_γδ complexes, with the latter lacking any endogenous ε and ζ subunits. The results showed that after the reconstitution of a large molar excess of the recombinant wild-type Pd-ε or mutant Pd-ε^ΔCT^ subunits, neither exerted any inhibition whatsoever in the steady-state PdF_1_-ATPase activity (Figure 4). As we observed previously with Pd-ε wild-type Pd-ε [29], the reconstitution of the Pd-ε^ΔCT^ recombinant subunit exerted a slight but reproducible activation of PdF_1_-ATPase both in the full PdF_1_-ATPase as well as in the Pdα_3_β_3_γδ complex. Once more, this is putatively explained by the stabilization of the Pdα_3_β_3_γδ and PdF_1_-ATPase complexes by substituting the missing endogenous Pd-ε subunit [29]. These results conclusively confirm the non-inhibitory nature of the Pd-ε subunit, regardless of its longer length. 

Therefore, the inhibitory function was transferred by evolutionary convergence from Pd-ε to Pd-ζ in α-proteobacteria, resulting in a fully unidirectional pawl/ratchet inhibition of the PdF_1_F_O_-ATPase activity [41,42,44,50]. We could explain this non-inhibitory character of Pd-ε by, for instance, some intrinsic incapacity to change its conformation from the non-inhibitory compact state to the extended inhibitory conformation. This is supported by our limited proteolysis and modeling data, which showed that the C-terminus of Pd-ε is accessible to trypsin in the PdF_1_-ATPase, and thus it is very likely in the compact conformation. Otherwise, the extended conformer of Pd-ε had occluded to the protease its C-terminus in the α_DP_/β_DP_/γ interface [41]. 

Taken together with the phylogeny, structural, and functional data, it becomes clear that ζ evolved to replace ε as the main inhibitor of the F_1_F_O_-ATPase of α-proteobacteria. In this context, we propose that according to the endosymbiotic theory [11,12,13] and the close relationship between mitochondria and α-proteobacteria [12], it is very likely that ε lost its inhibitory capacity in α-proteobacteria before the mitochondrial endosymbiotic event, thus leading to the actual mitochondrial non-inhibitory δ subunit and the evolutionary pressure to promote the appearance of a new mitochondrial F_1_F_O_-ATPase inhibitor or IF_1_. This implies that the non-inhibitory ε subunit of α-proteobacteria may be used as a tracer gene of the α-proteobacterial pre-endosymbiont from which mitochondria arose.

### 3.2. The In Vivo Roles of the Mitochondrial F_1_F_O_-ATPase (IF_1_), Bacterial ε, and α-Proteobacterial ζ Subunits

It was in mitochondria where the first supernumerary F_1_F_O_-ATPase inhibitor was discovered and named “IF_1_”, or intrinsic inhibitor of the mitochondrial F_1_-ATPase [15]. Subsequently, in bacteria such as *E. coli*, it was found that the essential subunit ε worked as a partial bacterial F_1_F_O_-ATPase inhibitor additionally to its primary role as F_1_-F_O_ connector in the central rotor [7]. In parallel, a DTT-sensitive disulfide bridge of the γ subunit was found to work as a redox-inhibitory domain of chloroplast F_1_F_O_-ATP synthases [16,17] besides also having the inhibitory ε subunit in CF_1_F_O_ [8,56,57,58,59]. We suggest keeping in mind that all these inhibitory proteins or domains work in most cases in addition to the insufficient MgADP partial inhibitory mechanism present in all F_1_F_O_-ATPases in nature. More recently, the pawl/ratchet unidirectional mechanism of the so-called ζ subunit of *P. denitrificans* and related α-proteobacteria was discovered and described in detail, with the latter, of course, having a close evolutionary relationship to the endosymbiotic origin of mitochondria [12]. We also found that the ζ, IF_1_, and ε subunits, and also the chloroplast γ-disulfide domain, share the same inhibitory final position in the α_DP_/β_DP_/γ interface, called INGECORE or (Inhibition General Core Region) [41,44,50], and this was confirmed with simultaneous crystallographic evidence [60]. Most likely, all of these inhibitory proteins, mitochondrial IF_1_, bacterial ε, and α-proteobacterial ζ, besides the chloroplast γ-disulfide domain, work as unidirectional pawl/ratchets, exclusively blocking their respective F_1_F_O_-ATPase activity associated with the CCW rotation of the nanomotor, without disturbing the CW rotary turnover of the F_1_F_O_-F-ATP synthase turnover [41,44,50]. In the case of the chloroplast γ disulfide bridge, it is reverted by thioredoxin and ferredoxin during illuminated photophosphorylation. This general ratchet mechanism of all these F-ATPase inhibitors provides an effective means to preserve the precious cellular ATP by blocking the detrimental CCW F_1_F_O_-ATPase rotation exclusively, while allowing the CW F_1_F_O_-ATP synthase turnover to go forward.

The key role of these natural F_1_F_O_-ATPase inhibitors has been researched by means of knockout or null IF_1_ and ζ mutants, given that it is not possible to delete the essential ε subunit since its deletion leads to the detachment of F_1_ from F_O_ in bacterial F-ATP synthase because of its primary function as a central rotor subunit connecting F_1_ with F_O_ [31]. Several knockouts of mitochondrial IF_1_ in fungi [61], yeast [62], nematodes [63], and mice [64] failed to show a knockout phenotype different to the wild-type strains, either because there are multiple copies of the IF_1_ gene or because all of these species contain assembly or stabilizing factors genes (STFs) similar to IF_1_ [65,66,67,68]. These IF_1_ knockouts have only been made in a single IF_1_ gene in most cases, and therefore, the other IF_1_ gene copies or IF_1_-like stabilizing factors (STF1 or STF2) very likely complement the missing IF_1_ gene in these single knockouts, thus explaining the lack of a clear different phenotype of the null mutants compared with the wild-type strains. Only one yeast study deleted all the IF_1_ and STF genes in a triple knockout mutant in *Saccharomyces cerevisiae*. In that case, a clear respiration-deficient phenotype different from the wild-type yeast was found that also showed an increase in the ATP-driven proton pumping in yeast mitochondria [62]. 

In summary, in eukaryotes, only in multiple-knockout mutants where all IF_1_ (or INH1) gene copies, together with STF1, STF2, and related IF_1_-like proteins, are deleted, a different mutant vs. wild-type phenotype is expected. Therefore, all single and double IF_1_ and/or STFs mutants already constructed do not answer the question of whether IF_1_ (or IF_1_-like proteins) have an important role in mitochondrial or cell bioenergetic metabolism or not. In contrast, taking advantage of the fact that in *P. denitrificans* there is only one copy of the ζ gene and no additional ζ-like genes, recently, we were able to report the first knockout mutant of any F_1_F_O_-ATPase inhibitor having a clear defective phenotype in its respiratory succinate growth after making the knockout of the ζ subunit in *P. denitrificans*, named the PdΔζ mutant. This mutant showed a drastically slowed down growth rate in respiratory succinate media, a decreased cellular ATP concentration (ATP), and a 2–6-fold increased ATPase activity coupled to enhanced proton release across the plasma membrane, as expected from knocking out an important F_1_F_O_-ATPase inhibitor [42,69]. More conclusively, these detrimental features of the PdΔζ knockout were reverted by complementation with a wild-type ζ gene of *P. denitrificans* expressed in trans [42,69]. 

Others have constructed another multiple PdΔHyΔζ mutant, but unfortunately, it also lacked eight hydrogenase genes additional to the ζ gene, thus being a complex construction with multiple deletions that resulted in a phenotype identical to the PdWT strain when grown in succinate media. This phenotype is most likely a result of complementation between the nine different deletions [70]. Their lack of a different phenotype after ζ deletion in their PdΔHyΔζ mutant led them to the conclusion that ζ is not the main inhibitor of the PdF_1_F_O_-ATPase. Instead, they propose that both MgADP and the Pd-ε subunit should have the main inhibitory roles [49,70]. However, our phylogenetic, functional, and structural evidence reviewed here indicates that they misinterpreted their results by not considering the phenotypic complementation between their multiple nine deletions in their PdΔHyΔζ mutant, because our single deletion of ζ has a strong effect on the respiratory growth of *P. denitrificans.* Furthermore, we also recently demonstrated that in minimal media during anaerobic conditions, the ζ subunit is not only important but *essential,* since the PdΔζ mutant does not grow at all. In contrast, the PdWT strain shows optimal growth in anaerobiosis (Sharon Rojas-Alcantar and José J. García-Trejo, unpublished). Similarly, regarding this Special Issue of adaptation to oxygen, we also observed that *P. denitrificans* modulates the ζ expression to adapt and optimize its growth in the presence or absence of oxygen, confirming the important role of ζ as the main regulatory protein of the PdF_1_F_O_-ATP synthase. Therefore, our PdΔζ mutant is the only Pd-ζ knockout suitable to obtain conclusive data on the in vivo function of ζ, given that it was constructed on the PdWT (Pd1222) background and no other genes were deleted or mutated, i.e., only the Pd-ζ gene was replaced by a kanamycin resistance cassette marker [42,69]. The fact that our PdΔζ knockout strain has strongly delayed growth in succinate shows two main facts: (1) because there is a single Pd-ζ gene copy in the chromosomal DNA of *P. denitrificans*, the biological role of the natural F_1_F_O_-ATPase inhibitors can be revealed more clearly in *P. denitrificans* than in the previous IF_1_ knockouts in yeast, mice, and fungi because in *P. denitrificans,* there are no other Pd-ζ-like genes or Pd-ζ gene copies that may complement the PdΔζ null mutant, as likely occurred with the previous IF_1_ knockouts. This implies that the mitochondrial IF_1_ should also have a key role in the prevention of wasteful mitochondrial F_1_F_O_-ATPase activity, and probably because of this key role of IF_1_, there are several copies of IF_1_ or IF_1_-like genes (STFs) in eukaryotes, perhaps in the case that one IF_1_ gene or protein may somehow fail in its expression or inhibition. (2) As explained above, the slowed-down growth of the PdΔζ mutant in respiratory media confirms that the intrinsic MgADP inhibitory mechanism preserved in most F-ATP synthases is insufficient to prevent wasteful F_1_F_O_-ATPase activity during the respiratory growth of *P. denitrificans* in succinate media. Therefore, the ζ subunit is essential to prevent and block most of the wasteful F_1_F_O_-ATPase activity in vivo. Suppose kinetic MgADP inhibition were more than enough to preserve the bacterial ATP. In that case, the PdΔζ mutant should have grown identically to the wild-type PdWT strain in respiratory media since the ζ would be dispensable if the MgADP inhibition of the PdF_1_F_O_-ATPase suffices for the bacterial respiratory physiology. However, since this is not the case, it is clear that the MgADP inhibition is insufficient, and therefore, the ζ subunit has the main role in preventing the wasteful PdF_1_F_O_-ATPase activity. In summary, the actual experimental and bioinformatics evidence demonstrates the key role of the ζ subunit as the major control or inhibitory mechanism in the PdF_1_F_O_-ATPase. Accordingly, the ζ gene is present in most if not all α-proteobacteria as an exclusive ORF in this important bacterial class. Thus, the inhibitory function of the ζ subunit needs to be confirmed in other α-proteobacteria related to *P. denitrificans*, which are more or less related to the endosymbiotic origin of mitochondria. So, we performed further functional and structural analyses to complement the evolutionary analysis of the ζ subunit across α-proteobacteria. 

### 3.3. The Function and Mechanism of the ζ Subunit of the α-Proteobacterial F_1_F_O_-ATPase

In contrast to the bacterial inhibitory subunit ε, or the newly discovered AtpΘ subunit, which always leave residual ATPase activity, the ζ subunit inhibits the F-ATPase activity totally. Some of us had shown this total inhibition by ζ in both “macro” standard PdF_1_-ATPase assays [29,41] and with unimolecular PdF_1_-ATPase rotary assays [71]. The latter was in collaboration with Prof. Hiroyuki Noji from the University of Tokyo. Particularly in the latter study, we showed that MgADP inhibition only exerts a partial inhibitory effect of unimolecular PdF_1_-ATPase rotation [71]. In contrast, the ζ subunit completely stalls the CCW rotation of the PdF_1_-ATPase nanomotor at the unimolecular level, thus explaining why ζ also exerts total inhibition in macro-PdF_1_-ATPase assays. This confirms the partial and insufficient inhibitory effect of MgADP and the total and, therefore, enough inhibition exerted by ζ. The inhibitory mechanism of this subunit has been described in detail by our group and coworkers, firstly discovering the ζ subunit and describing its intrinsically disordered inhibitory N-terminal domain, subsequently demonstrating that ζ and IF_1_ share the same inhibitory binding site at the α_DP_/β_DP_/γ interface of the F_1_-ATPase [14,29,30,41,51,72]. Afterwards, our null or PdΔζ knockout mutant was the first F_1_F_O_-ATPase inhibitor that showed a clear phenotypic difference between the wild-type and null mutant strains [42,44,50]. As explained before in the latter references and above, all other null mutants of IF_1_ and the PdΔHyΔζ of Hirst and coworkers failed to have a different phenotype to the PdWT strain. Further details on the structure and function of ζ has been reviewed before [42,44,50]; thus, we focus on the key point here, which is that our PdΔζ mutant is the first F_1_F_O_-ATPase inhibitor knockout that demonstrates the important role of the ζ subunit for *P. denitrificans* in respiratory media, and therefore strongly suggests a similar key role for the other two classical F_1_F_O_-ATPase inhibitors, namely inhibitory bacterial ε and mitochondrial IF_1_ proteins [42,44,50]. These studies also demonstrated that the ζ subunit works as a unidirectional pawl/ratchet inhibitor [41] exclusively blocking the counterclockwise (CCW, viewed from F_O_ to F_1_) rotation of γ at the central rotor during PdF_1_F_O_-ATPase activity, but allowing the progress of clockwise PdF_1_F_O_-F-ATP synthase turnover [41,42,44,50]. This pawl/ratchet mechanism is optimal to protect the ATP pools of the cell and, therefore, improve the overall cellular bioenergetics. By combining the crystallographic PdF_1_F_O_-ζ structure (PDB_id 5DN6) with our soluble Pd-ζ NMR structure (PDB_id 2LL0) [42], we also found that this globular and α-helical bundle domain of Pd-ζ works as an anchoring domain interacting mainly with the β subunit of the PdF_1_F_O_-ATPase [42,44,50]. These structural features explain the strong conservation of the N-termini of the ζ subunits along with the α-proteobacteria class and the high variation or divergence of the Pd-ζ C-terminal and globular domain, which should adapt to co-evolving and species-specific mutations in the C-termini of β and perhaps α subunits where ζ is anchored [42,44,50].

### 3.4. Distribution of the ζ Subunit along the α-Proteobacterial Class: From Domain of Unknown Function DUF1476 to αPATPζ

Our bioinformatic results confirmed, once more, that the ζ subunit is essentially a protein family (DUF 1476) exclusive to the α-proteobacteria class. Because the genomics and classification of α-proteobacteria has been recently updated [73], we also updated and confirmed the strict conservation of the functional N-terminal domain of the ζ subunit family and the increasing gradual divergence of the ζ protein family going from the inhibitory N-terminus towards the C-terminus, as we observed before [29]. 

The gene that encodes for the ζ subunit has been proposed to be called AtpZ [74]. However, the ζ gene is not part of either of the two ATP operons that encode the F_1_ and F_O_ parts of the α-proteobacterial F-ATP synthase. The AtpZ gene described by Zavarzina et al. is not the gene of the ζ subunit, but it is indeed a gene similar and contiguous to ATPI, the assembly factor or chaperone of the *c* subunits of the ATP F_O_ operon. Therefore, here, we suggest naming the ζ gene as “αPATPsζ” (α-proteobacterial F-ATP synthase ζ gene); it is currently annotated as DUF1476 in different databases such as KEGG, PATRICK, and NCBI. We do not call it “ATPζ” to emphasize that this gene is not part of either of the two ATP (previously *UNC*) operons of α-proteobacteria. We found that this αPATPsζ gene is present essentially and exclusively in α-proteobacteria. A few hits external to the α-proteobacteria class are truncated pseudogenes likely transferred horizontally to other bacterial classes. As a result, ζ assumes higher physiological and evolutionary significance since the F_1_F_O_-ATPase from α-proteobacteria is more closely related to its mitochondrial counterpart than to F-ATP synthases from other bacterial phyla and classes [41].

It is generally accepted that the F_1_F_O_-ATPase of the α-proteobacteria is a direct predecessor of the mitochondrial enzyme because the pre-endosymbiont that evolved into the actual mitochondria most likely came from an ancestral α-proteobacteria, in concordance with the endosymbiotic theory promoted by Lynn Margulis [12] and more recent genomic analyses [75,76]. Several years ago, *Paracoccus denitrificans* was considered closely related to the free-living ancestor of the actual mitochondria [77,78,79,80], and it was even considered a microorganism similar to a free-living mitochondrion [81]. However, more recently, it is well accepted that other α-proteobacteria, such as those in the Rickettsiales order, might be phylogenetically closer to the actual mitochondria [82]. 

Accordingly, our modeling of the PdF_1_-ATPase was constructed to resolve the Pd-ζ subunit-binding site used as a template for the structure of mitochondrial F_1_-ATPase and not the bacterial one, since the PdF_1_-ATPase has a higher identity with the mitochondrial enzyme than with other bacterial F_1_-ATPases such as that of *E. coli* [41]. The main difference between the mitochondrial and α-proteobacterial F_1_F_O_-ATP synthases is that the mitochondrial enzyme has additional supernumerary accessory subunits involved in the dimerization of the mtATP synthase, cristae formation (see below), and the inhibitory IF_1_ subunit. As we see later, IF_1_ is the analog to the ζ subunit in α-proteobacteria. The structure of IF_1_ is one extended α-helix (PDB_id 1GMJ), whereas the structure of the ζ subunit is a four-α-helix bundle (PDB_id 4LL0). Given these differences between ζ and IF_1_, we analyzed whether ζ and IF_1_ are homologs, either paralogs that could have coexisted in α-proteobacteria and mitochondria, or orthologs separated by speciation before, during, or after the mitochondrial endosymbiosis. 

**Figure 5:**
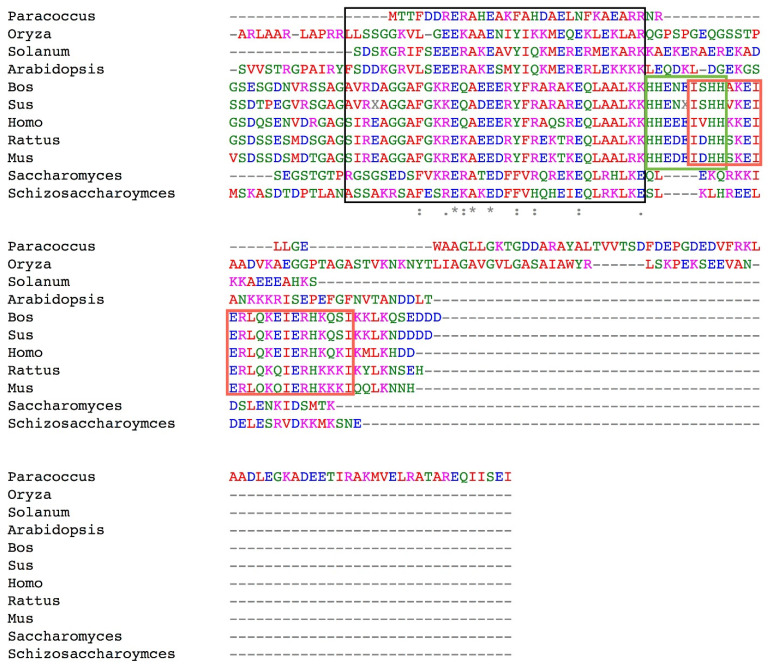
**Alignment of the ζ subunit of *P. denitrificans* (Pd-ζ) with the inhibitory mitochondrial IF_1_ protein family.** The full sequence of Pd-ζ was aligned with representative sequences of mitochondrial IF_1_ inhibitor proteins from yeast, animals, and plants. The IF_1_ sequences correspond to the mature proteins, with the first residue starting to alter removal of the mitochondrial targeting sequences (MTSs). The region boxed in black (☐) corresponds to the N-terminal part of the minimal inhibitory sequence of IF_1_ as defined by biochemical [91] and genetic deletion analyses [92] and by direct observation of contact residues between IF_1_ and the F_1_-ATPase in crystal structures [93]. (*) indicates identical residues, (:) show very similar residues, and (.) indicates similar residues. Blank marks ( ) show variable residues following Clustal-Ω alignments. The green box indicates the segment containing the pH sensor of His pairs in animal IF_1_s (☐). The red-orange box (☐) shows the homodimerization domain of animal IF_1_s with the Ile heptads in the C-termini that promotes IF_1_-IF_1_ and F_1_F_O_-F_1_F_O_ dimerization, tetramerization, further mtATP synthase oligomerization, and mitochondrial cristae formation (see text and Figure 6 and Figure 7). We previously presented a simpler version of this alignment [29]. Aligned species are *Paracoccus denitrificans*, representing the ζ subunit, and the rest of the sequences correspond to mature mitochondrial IF_1_s: *Oryza sativa*, *Solanum tuberosum*, *Arabidopsis thaliana*, *Bos taurus*, *Sus scrofa*, *Homo sapiens*, *Rattus norvegicus*, *Mus musculus*, *Saccharomyces cerevisae*, and *Schizosaccharoymyces pombe*. See text for further details.

Even though the N-terminal inhibitory domains of ζ and IF_1_ are similar enough to promote their productive binding to the same α_DP_/β_DP_/γ interface in their F_1_-ATPases [41,44,50], their sequence identity is too low to be considered homologs. The alignment of both full mature subunits in the cases of Pd-ζ and several mitochondrial IF_1_s from *Homo sapiens*, *Bos taurus*, *Sus scrofa*, *Rattus norvegicus*, *Saccharomyces cerevisiae*, *Arabidopsis thaliana*, and *Oryza sativa*, among other eukaryotes, showed that their identity is on average only ≤3.5%. Given the slight similarity of the inhibitory N-termini of ζ and IF_1_ (see Figure 5 and Supplementary Material of Ref. [29]), the overall similarity between them is only about 11%. Our previous alignment of the Pd-ζ N-terminus with the same IF_1_s was carried out only with the N-terminal inhibitory sequence of the first 17 a.a.s of Pd-ζ and the N-terminus of IF_1_s (see Supplementary Material of Ref. [29]). Therefore, here, we improved this alignment by carrying it out with the full mature ζ and IF_1_ sequences, as shown fully in Figure 5. This was a key result because the low identity and similarity values between ζ and IF_1_ fall far below the consensus 30% identity threshold to consider ζ and IF_1_ as putative homologs. So, the overall structures of ζ and IF_1_ are, in essence, mostly different; thus, it is very likely these two F_1_F_O_-ATPase inhibitors are analogs rather than homologs. The ζ subunit was not even closer to the IF_1_s of unicellular eukaryotes such as yeast but could only be aligned to the IF_1_ of rice (*Oryza sativa*). Once again, the only similar region between ζ and the several IF_1_s coincided exactly with the inhibitory N-terminal and intrinsically disordered protein region (IDPr) of both proteins that fold into an α-helix upon productive binding to their respective F_1_-ATPases, as we described before [29] (see a black box in Figure 5). As can be seen in this Pd-ζ vs. IF_1_s alignment, the ζ subunit is much larger than all IF_1_s, and this extra C-terminal extension provides ζ with a larger and different anchoring domain than IF_1_. Therefore, these data may respond to the question that arises as to why two different inhibitory subunits regulate the F_1_F_O_-ATPases from α-proteobacteria and mitochondria. By answering this question, we could shed light on identifying the microorganism of the α-proteobacteria that was closer to the pre-endosymbiont and evolved into the actual mitochondria, a matter that is still very much under debate [83,84,85,86,87,88,89,90]. 

Once we identified that ζ and IF_1_ are analogs rather than homologs, we searched for the presence or absence of the ζ gene in eukaryotes and the presence or absence of the IF_1_ gene in prokaryotes, particularly in α-proteobacteria. The mutual absence of ζ in eukaryotes and IF_1_ in prokaryotes would confirm that ζ and IF_1_ emerged and evolved independently as analogous rather than homologous proteins.

Blasting the ζ gene in Eukarya revealed only two hits, the first of a mycoparasite *Syncephalis pseudoplumigaleata* (*S. pseudoplumigaleata*), which lives as a parasite of other fungi in the soil. The second was *Symbiodinium microadriaticum* (*S. microadriaticum*), a dinoflagellate microalga that is an intracellular endosymbiont of corals and anemones, jellyfish, demosponges, flatworms, and mollusks. In the first case, *S. pseudoplumigaleata*, the apparent homologous ζ gene seems to be a putative biosynthetic protein of purines (NCBI Sequence ID: RKP2829.1), which are synthesized in the cytosol.

In the second case, *S. microadriaticum*, it is a DUF (Domain of Unknown Function) or unnamed protein product (Sequence ID: CAE7256085.1). It is well known that α-proteobacteria can inhabit soil and seawater, as in the case of *P. denitrificans* and *Oceanicola* sp., or *Jannaschia* sp., respectively. In addition, some of these α-proteobacteria are also intracellular parasites or facultative symbionts. Therefore, the two unique eukaryotes harboring a ζ-like gene could result from horizontal transfer between a donor α-proteobacteria, perhaps symbiont or parasitic, and an acceptor such as *S. pseudoplumigaleata* or *S. microadriaticum*. Because these are the only two cases where a putative homologous ζ subunit exists in eukaryotes, it seems proper to conclude that the ζ gene is essentially absent in the eukaryotic chromosomal and mtDNA.

We also searched for the presence or absence of IF_1_ in bacteria, particularly in α-proteobacteria. In the former, we only found two blast hits corresponding to hypothetical proteins of two enterococci. The first one was the hypothetical protein DKP78_15735 of *Enterococcus faecium* (sequence ID: PWS22945.1), and the second was a hypothetical protein of *Enterobacter cloacae* complex sp. 2DZ2F20B (sequence ID: WP_129335269.1). Both enterococci could be commensals or pathogens in humans and other animals and, therefore, could have acquired the animal IF_1_ gene by indirect horizontal transfer from the animal or human hosts. In α-proteobacteria, the IF_1_ gene was absent since Blast did not find any hit. In summary, because the ζ and IF_1_ subunits were reciprocally absent in eukaryotic and prokaryotic organisms, respectively, this strongly suggests that ζ and IF_1_ are neither paralogous nor orthologous genes. Therefore, we suggest that ζ and IF_1_ are analogous products of evolutionary convergence. 

In consequence, if we could identify some α-proteobacteria that lack the αPATPsζ gene in their genomes, this could help us to understand why there is no ζ subunit in eukaryotes and why the IF_1_ inhibitory subunit is absent in α-proteobacteria and therefore arose after the mitochondrial endosymbiosis. The α-proteobacteria that had lost the αPATPsζ gene in their genomes might be ideal candidates to be the closest organisms to the mitochondrial pre-endosymbiont.

### 3.5. The ζ Subunit Gene Is Absent in the Acetobacteraceae and Holosporaceae Families and in the Order Rickettsiales of α-Proteobacteria

In order to assess if some α-proteobacteria had lost the ζ subunit gene, in this work, we carried out a systematic search of the αPATPsζ gene in all orders, families, and genera of α-proteobacteria. We found that the αPATPsζ gene is not present in all α-proteobacteria but is absent in some important members of this class. We identified three major groups where the αPATPsζ gene is absent: the family Holosporaceae from the order Holosporales, which has 27 genomes sequenced, and the family Acetobacteraceae from the order Rhodospirillales, and the whole order Rickettsiales. Interestingly, the Acetobacteraceae family has 49 genera, with 1371 genomes sequenced. The blast result for ζ rendered only four hits, indicating that this F-ATP synthase subunit is absent from this major group. In the Holosporaceae family of the Holosporales order, there were no significant hits of the ζ subunit gene or DUF1476 domain. In the order of the Rickettsiales, there are 2121 genomes sequenced, which consist of three major families: (1) Anaplasmataceae (1488 sequenced genomes), (2) Candidatus Midichloriaceae (30 genomes sequenced), and (3) Rickettsiaceae (376 genomes sequenced), the blast of the ζ subunit rendered only 12 hits with the ζ gene. This clearly indicates that the ζ subunit gene is absent in most of the genomes of this order. It is possible that the ζ positive hits in the Acetobacteraceae family or the Rickketsiales may show α-proteobacteria that, for some reason, still preserve the ζ subunit. However, these positive hits may also result from wrong annotations, contaminated samples, or possible horizontal transfer, so it seems suitable to conclude that the ζ gene is essentially absent in the whole Rickettsiales order and also in the Acetobacteraceae and Holobacteraceae families. As suggested, the lack of the αPATPsζ gene in these α-proteobacterial orders and families may have important implications in the search for the identity of the mitochondrial pre-endosymbiont that evolved into the present mitochondria.

In a recent work simultaneous to this review [84,94], we also studied the evolution and function of the ζ subunit across the α-proteobacteria class firstly by similar bioinformatic analyses of the α-proteobacterial ζ subunit, and in parallel by molecular cloning and biochemical and structural analyses of some ζ subunits and their corresponding F-ATP synthases in distinct bacterial families. The latter included several types of α-proteobacteria, such as strictly free-living respiratory *Paracoccus denitrificans* or photosynthetic *Rhodobacter sphaeroides* (now Cereibacter sphaeroides), facultative symbiotic *Sinorhizobium meliloti*, facultative parasitic *Brucella canis*, and strictly parasitic *Wolbachia pipientis* α-proteobacteria [94]. To accomplish a wider evolutionary analysis of the ζ subunits of several α-proteobacteria from different orders, we cloned and over-expressed some recombinant ζ subunits from the aforementioned α-proteobacteria. With these recombinant ζ subunits, we carried out several homologous or heterologous reconstitutions of the various ζ subunits to their respective α-proteobacterial F_1_ or F_1_F_O_-ATPases. These results indicated that the evolution of the ζ subunits was in accordance with different bacteria’s functional and energetic requirements. For instance, the inhibitory capacity of ζ is preserved in free-living and thus more environmentally challenged α-proteobacteria. On the other hand, this inhibitory function is reduced in some facultative symbiotic α-proteobacteria. Finally, we found that the αPATPsζ gene was totally lost in strictly parasitic or Rickettsial α-proteobacteria, among others, with the latter, of course, less challenged by environmental changes. This evolutionary pattern is in concordance with the major bioenergetic requirement of ATP synthesized by the α-proteobacterial F-ATP synthase in the free-living α-proteobacteria, rather than in facultative symbiotic and strictly parasitic α-proteobacteria which may obtain nutrients and/or ATP directly from their hosts. Furthermore, we resolved and correlated the structure of the ζ subunit from *Synorhizobium meliloti* with its lack of homologous inhibitory function. The results showed a pattern in which the evolution of the ζ protein is linked to its function and the selective pressure exerted by the metabolism and lifestyle or environment of the respective α-proteobacteria [94]. The ζ subunit preserves its inhibitory function in free-living α-proteobacteria such as the denitrifying *P. denitrificans* and the photosynthetic *R. sphaeroides* (Jorge Brito-Sánchez and José J. García-Trejo, unpublished) but lost part of its inhibitory capacity in *Sinorhizobium meliloti*. In contrast, the αPATPsζ gene and ζ protein were completely lost in some strictly parasitic α-proteobacteria such as the order of Rickettsiales [94]. This is an important finding because the ζ subunit evolved, losing its inhibitory potency and ultimately its presence when it could be partially or totally dispensable. In other words, in free-living α-proteobacteria, ζ is indispensable probably because of the wider environmental changes experienced by free-living α-proteobacteria to prevent wasteful hydrolysis of the valuable ATP pools. On the other hand, in facultative symbiotic bacteria such as *S. meliloti*, the inhibitory function was partially lost, given that the ζ subunit of *S. meliloti* (Sm-ζ) is not able to inhibit its own SmF_1_-ATPase. However, it can exert heterologous inhibition on the PdF_1_F_O_-ATPase [94]. Therefore, the Sm-ζ preserves some inhibitory potential. Structurally, we found that this lack of homologous inhibition by Sm-ζ is associated with a different and ordered structure of the inhibitory N-terminal domain of the Sm-ζ compared to its homologous inhibition from *P denitrificans* (Pd-ζ) [94]. Accordingly, these facultative symbiotic Rhizobiales bacteria may exchange nutrients and be in less stressful conditions during symbiotic growth, thus making the ζ subunit less essential but still preserving the αPATPsζ gene and ζ protein as a putative emergence use in hard environmental or low intracellular ATP conditions that may occur in the shift from a symbiotic to free-living lifestyle. We do not discard the concept that the ζ subunit could have another function besides inhibiting the α-proteobacterial F_1_F_O_-ATPase, thus explaining why the αPATPsζ gene and ζ protein are preserved in Rhizobiales or in other orders where its inhibitory capacity could be diminished. For instance, it has been described in the case of *Zymomonas mobilis* (order: Sphingomonadales, family: Zymomonadaceae) that the overexpression of this αPATPsζ gene (identified as ZMO1875 or DUF1476) improved the ethanol productivity in the presence of hydrolysate [95]. The authors propose that the αPATPsζ gene has moonlight functions in this bacterium in the assembly of Fe-S clusters or in their de novo biosynthesis, perhaps besides inhibiting the F_1_F_O_-ATPase.

**Figure 6:**
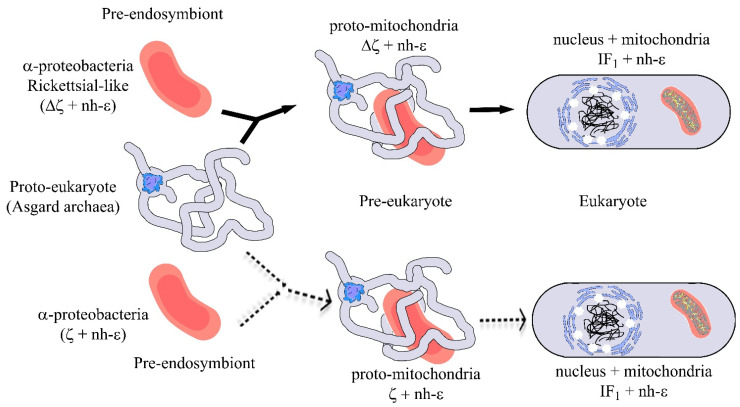
**Simplified mitochondrial endosymbiotic events with an α-proteobacterial pre-endosymbiont having or lacking the ζ gene.** The upper part (black arrows) shows the most likely mitochondrial endosymbiotic event, assuming that ζ is not a homolog of IF_1_, with a Rickettsial-like pre-endosymbiont lacking the αPATPsζ gene (Δζ), which evolves within a pre-eukaryote from being an intracellular parasite into a proto-mitochondria lacking ζ and already having a non-inhibitory ε subunit (nh-ε) in its F-ATP synthase. This eventually transforms into mitochondria in an enucleated eukaryote. In this more likely scenario, ζ and IF_1_ arose separately by convergent evolution. In mitochondria, IF_1_ evolved not only to preferably inhibit the F_1_F_O_-ATPase activity, but also to stabilize the mitochondrial F-ATP synthase dimers, and further oligomers that give its shape to the cristae of the mitochondrial inner membrane (see yellow spots of F-ATP synthases in the curved portions of cristae and Figure 7). The lower part (dashed light arrows) shows a less likely alternative scenario where ζ is the predecessor of IF_1_ and where an α-proteobacterial pre-endosymbiont having ζ and containing an nh-ε became a protomitochondria in the pre-eukaryote, and this evolved to become IF_1_ in mitochondria of the nucleated eukaryote. This alternative is less likely because of the low identity between ζ and IF_1_ and because there is no evidence of the presence of IF_1_ genes in α-proteobacteria or other bacteria, and the other way around, there is no trace of ζ genes in eukaryotes. See text for further details. Figure adapted from Ref. [96].

In strict intracellular parasites such as the whole Rckettsiales order, these bacteria are very dependent on their hosts since they consume the cytosolic ATP from the host’s cells. This makes the αPATPsζ gene totally dispensable, to such a degree that the αPATPsζ gene was totally lost in some of these strictly parasitic α-proteobacteria. This Rickettsiales order closely resembles the eukaryotic mitochondrial DNA in their chromosomal DNA [82], strongly suggesting that some of these Rickettsial-like α-proteobacteria lacking the αPATPsζ gene could have become the mitochondrial pre-endosymbiont because of its strictly parasitic lifestyle (see Figure 6). 

However, it is possible that α-proteobacteria from other orders could harbor the pre-endosymbiont that evolved into mitochondria. For instance, it has also been suggested that the mitochondrial pre-endosymbiont may have emerged from Rhizobiales or Rhodobacterales [97]. In this regard, we found that some Rhizobiales, such as *Synorhizobium meliloti*, had lost the homologous inhibitory capacity of their own F-ATP synthase’s ζ subunit due to structural changes in their N-terminal sides [94]. Therefore, it could be considered that the mitochondrial pre-endosymbiont may already have had a non-inhibitory ζ subunit such as that of *S. meliloti* of the Rhizobiales order. Therefore, this dysfunctional ζ gene could have vanished during the endosymbiotic evolution and gene transfer process, transforming the initial proto-mitochondrion into the actual mitochondria. This would be a hybrid process combining the upper and lower scenarios of Figure 6, which is supported by the close position of the Sm-ε subunit to the mitochondrial δ subunit (around 5’o’clock in the ε cladogram of Figure 3). Other possibilities derived, for instance, from the ε cladogram of Figure 3 are that some α-proteobacterial ε subunits closer to the mitochondrial δ subunit are those of the Hyphomicrobiales (*Rhodopseudomonas palustris)*, Caulobacterales (*Caulobacter mirabilis*), or Rhodospirillales (*Rhodospirillum rubrum*), among others. However, further comparative phylogenetic analyses with other more conserved F-ATP synthase subunits should shed light on these possibilities on the origin of mitochondria from α-proteobacteria. We should also keep in mind that the closest relative to the mitochondrial pre-endosymbiont might be yet to be described, since new species of α-proteobacteria are being found [98] and new updated classifications of α-proteobacteria are still being described [73]. Coincidentally, in one of the latest phylogenetic studies of α-proteobacteria, Cevallos and Degli-Esposti found the Rickettsiales and Holosporales orders closer to the origin of mitochondria [98]. Accordingly, here, we found that the ζ subunit gene was lost in exactly the same α-proteobacterial orders (see above), suggesting that the endosymbiotic origin of mitochondria might be putatively traced to these two α-proteobacterial orders. These recent and oncoming phylogenetic results might work as a guide to look for the origin of mitochondria within the α-proteobacteria. In sum, the overall data reviewed here show that ζ is not an evolutionary predecessor or homolog of mitochondrial IF_1_, but rather, both inhibitor proteins emerged separately by convergent evolution. Taken together, these results strongly suggest that the absence or presence of the αPATPsζ gene, besides the non-inhibitory ε subunit (nh-ε), can be considered from now on as instrumental tracers in the search for the elusive mitochondrial pre-endosymbiont (Figure 6).

### 3.6. Post-Mitochondrial Endosymbiosis Epilogue: Mitochondrial IF_1_ Stabilizes the Dimerization and Oligomerization of the mtATP Synthase Besides Inhibiting the F_1_F_O_-ATPase

Once the mitochondrial endosymbiosis was established, a lot of gene transfers from mitochondria to the eukaryotic nucleus had been taking place in the last millions of years of biological evolution. For instance, it is clear that several subunits of the mitochondrial F_1_F_O_-F-ATP synthase are encoded in the eukaryotic nucleus. Only some genes of the F_O_ domain remain in the mitochondrial DNA (mtDNA), i.e., all the mitochondrial F-ATP synthase genes of the F_1_ and side stalk domains are transcribed in the nucleus, translated in the cytosol and imported into the mitochondria to participate in the biogenesis and assembly processes of the mitochondrial F-ATP synthase (mtATP synthase). The progressive mtDNA gene transfer to the nucleus is evidenced in the case of the mitochondrial F-ATP synthase by the fact that the three F_O_ genes are preserved in yeast mtDNA such as *Saccharomyces cerevisiae*; for the subunits 6 (or *a*), 8 (or A6L), and subunit 9 (or *c*), the respective mtDNA genes are ATP6, ATP8, and ATP9. On the other hand, in more recent eukaryotes such as mammals, and as in the case of the human mtDNA, the only two F-ATP synthase genes preserved are those of subunits six and A6L (ATP6 and ATP8), i.e., the subunit *c* gene (ATP9) has migrated from mtDNA to the nucleus in the evolution from yeast to mammals. It is evident that the mtDNA and chromosomal DNA of eukaryotes continue to evolve to adapt the F-ATP synthase to the requirements and challenges imposed by the mitochondrial metabolism within each cell type and species. For instance, the bacterial F-ATP synthase keeps a monomeric state as the functional unit, either as F_1_F_O_-ATPase or as F_1_F_O_-F-ATP synthase. This was first demonstrated by Blue-Native gel electrophoresis (BN-PAGE) of the inner membranes of *P. denitrificans* compared with the same electrophoretic analysis of mitochondrial membranes [99], and we confirmed these observations; the BN-PAGE of inner membranes of *P. denitrificans* show a single F-ATP synthase band corresponding to monomeric F-ATP synthase. In contrast, mtATP synthase always shows dimeric, tetrameric, and further oligomeric aggregation states (see for instance Ref. [14]).

**Figure 7:**
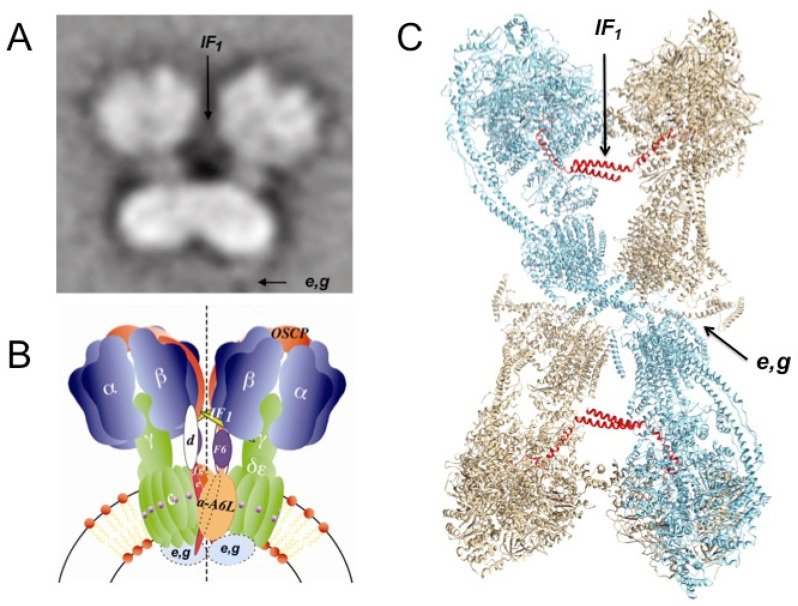
**Double function of mitochondrial IF**_1_**as intrinsic F_1_F_O_-ATPase inhibitor and as dimerizing and oligomerizing factor of the mitochondrial F-ATP synthase**. (**A**) The first EM 2D structure of bovine mitochondrial dimeric F_1_F_O_-IF_1_ complex [102] is shown as an average of the complete set of collected images, indicating the originally proposed sites for IF_1_ as a central protein bridge structure connecting the F_1_–F_1_ moieties. On the other hand, the proposed dimerizing subunits e and g form a “hanging bridge” at the Fo-Fo dimer interface [102]. (**B**) Two-dimensional cartoon model of the mitochondrial F-ATP synthase dimer showing the proposed positions of IF_1_ (yellow) and e and g subunits (red/blue) according to functional and mutagenesis studies [103,104,105,106,107]. (**C**) The structure of tetrameric mitochondrial F-ATP synthase of pig *Sus scrofa* as resolved by Cryo-EM depicted as two small-angle (≈40°) V-shaped homodimers bridged by an antiparallel coiled-coil homodimer of the IF_1_ inhibitor (red). Two large-angle dimers (≈60°–90°) form another, more open V-shaped dimer connected through F_O_-F_O_ dimerizing subunits (e, g, and others) at the center of the tetramer. The dimerizing and oligomerizing function of mitochondrial IF_1_ lies on its C-terminal domain forming an antiparallel IF_1_-IF_1_ coiled-coil (red F_1_-F_1_ bridge and see Figure 1D and Figure 7), and its inhibitory domain is inserted in the α_DP_β_DP_γ interface of each monomer (see Figure 1C). The dimerizing function of IF_1_ was also demonstrated functionally by some of us with removal and reconstitution of IF_1_, which promoted, respectively, monomerization and dimerization/oligomerization of the mitochondrial rat liver F-ATP synthase. See Ref. [108] and text for further details.

On the other hand, the mtATP synthase has had to evolve and adapt its quaternary structure to form dimers and further oligomers to stabilize the formation of cristae of the inner mitochondrial membrane, thus increasing the membrane surface to improve the efficiency of oxidative phosphorylation [100,101,102]. This dimerizing and oligomerizing process involves the incorporation of several mitochondrial-specific dimerizing and oligomerizing subunits from the nucleus, mainly added to the mitochondrial F_O_ domain. Among these mtATP synthase’s dimerizing subunits are g and e, in addition to j and k, which provide most of the dimerizing F_O_-F_O_ interface (see Refs. [103,104,105,106,107] and Figure 1 and Figure 7).

At the beginning of the structural analyses of the dimeric mtATP synthase, some of us resolved the first 2D electron microscopic structures of the dimeric mtATP synthase, resolving a “small-angle” V-shaped dimer (≈45–50° angle) showing two protein bridging interfaces, one at the F_O_-F_O_ interface in the form of a “hanging” proteic bridge [102]. 

We also described another protein bridge at the F_1_-F_1_ interface [102]. In addition, some of us also provided functional reconstitution evidence that the removal and reconstitution of IF_1_ promoted the monomerization or dimerization/oligomerization of the mtATP synthase [108]. Others previously showed that IF_1_ dimerizes itself and also dimerizes the mitochondrial F_1_s [93,109,110,111]. Therefore, we proposed that the central F_1_-F_1_ bridging protein domain should contain the mitochondrial IF_1_-IF_1_ homodimer, stabilizing both the F-ATP synthase dimer and oligomer, and we also proposed that the F_O_-F_O_ “hanging” bridge should harbor the dimerizing e and g subunits (see Refs. [102,108] and Figure 7).

A large blast of the structural cryo-EM structures of dimeric mtATP synthase from several sources emerged since then and resolved a different dimeric structure of a large-angle V-shaped F-ATP synthase dimer (≈60–90° angle) which is present along the edges of mitochondrial cristae (see Refs. [112,113,114] and Figure 7), and therefore, our first 2D and 3D dimeric small-angle F-ATP synthases structures were considered by others as “artifacts” of our mitochondrial solubilization or dimer isolation procedures [112,113,114]. 

However, a recent cryo-EM structure of the tetrameric pig (*Sus scrofa*) mtATP synthase showed the first resolution of the bridging F_1_-F_1_ and F_O_-F_O_ protein interfaces at the same time [115]. Interestingly, they resolved two types of dimerizing interfaces, one forming the “large-angle” V-shaped F-ATP synthase dimer observed by the most recent cryo-EM analyses [112,113,114], and another “small-angle” V-shaped F-ATP synthase dimer, both of them forming the tetramer and promoting further oligomerization to form the mtATP synthase oligomer that shapes mitochondrial cristae.

Here, we propose calling the “large-angle” dimer the “Large-Angle Mitochondrial ATP synthase Dimer or LAMASD” and the “small-angle” dimer the “Small-Angle Mitochondrial ATP synthase Dimer or SAMASD” because both entities are present and important for mtATP synthase oligomerization and cristae formation. Comparing the pig mitochondrial SAMASD with our first 2D EM structure of the bovine mitochondrial F-ATP synthase dimer, it is very clear that we isolated and mostly resolved the “small-angle” lateral dimer, or SAMASD, in most of our EM analyses. However, we also observed the LAMASD dimer but with a lower frequency or stability in the yeast mtATP synthase dimer [116]. This preferred distribution of SAMASD mtATP synthase dimers in our preparations is likely the result of having a large concentration of MgADP, which is known to preserve the productive binding of IF_1_ to the mtATP synthase [15]. In contrast, in the dimer isolation and preparation by others, MgADP was mostly absent or not added to the dimer isolation media [112,113,114]. In consequence, we preferably isolated the SAMASD with a smaller angle of ≈45–50° bridged in the F_1_-F_1_ interface by the IF_1_-IF_1_ homodimer (Figure 7). The only difference between our 2D SAMASD and that of pig mtATP synthase tetramer is the disposition of the side stalks, which are more peripheral in the latter, and more central in our bovine or yeast SAMASD mtATP synthase dimers [115]. However, again this may also be the result of the presence of excess MgADP which promotes the formation of the IF_1_-IF_1_ inhibited MgADP state, whereas the resolved pig tetramer shows one F-ATP synthase monomer in the ADP state and the other monomer in the open or empty state [115]. Regardless of these differences, the conclusion is that our first 2D and 3D mtATP synthase dimers are clearly not artifacts but actual physiological and functional mtATP synthase dimer structures that form the SAMASD. This lateral mtATP synthase dimer of the oligomeric mtATP synthase shapes mitochondrial cristae together with the LAMASD. In consequence, the position of dimeric IF_1_-IF_1_ at the F_1_-F_1_ protein bridge and e and g subunits at the F_O_-F_O_ “hanging” protein bridge were properly predicted in spite of the low resolution of these primary dimeric mtATP synthase EM analyses [102].

Our previous functional observation of the role of IF_1_ in stabilizing the dimeric and oligomeric structures of the mtATP synthase [108], besides inhibiting the F_1_F_O_-ATPase activity, has been therefore structurally confirmed by the cryo-EM structure of the pig tetrameric F-ATP synthase [115]. Other IF_1_ knockout analyses have apparently shown that IF_1_ is not essential or necessary for the dimerization or oligomerization of the mitochondrial F-ATP synthase since the F-ATP synthase dimer is preserved in BN-PAGE analysis in both WT and ΔIF_1_ (or ΔINH1) knockout mutants [61,63,64,117]. However, as discussed above, at least one IF_1_ gene copy or homologous IF_1_-like gene that still remains expressed could complement or substitute the inhibiting and dimerizing function of the missing IF_1_. Therefore, the available IF_1_ knockout mutants do not demonstrate conclusively that IF_1_ or IF_1_-like proteins do not participate in mtATP synthase dimerization, so these are not the best experimental models to resolve this issue with. Rather, multiple mutants removing all IF_1_ and IF_1_-like proteins simultaneously will resolve the role of IF_1_ and its homologs in F-ATP synthase dimerization and cellular bioenergetics in eukaryotes. Furthermore, the recent cryo-EM structural resolution of the mtATP synthase tetramer [115] as well as our previous functional IF_1_ removal/reconstitution and BN-PAGE studies [108] confirm that IF_1_ exerts this important role in stabilizing the dimeric and oligomeric structure of the mtATP synthase that shapes mitochondrial cristae. 

The dimerizing domain of IF_1_ is an antiparallel coiled-coil segment in the C-terminal side of IF_1_, harboring the characteristic hydrophobic residue heptads that induce antiparallel coiled-coils (see Figure 5) [109,110]. This domain is absent in the other F-ATP synthase inhibitors, i.e., the α-proteobacterial ζ and bacterial ε subunits (see Ref. [29], Figure 1, Figure 4 and Figure 5), which show very different structures in their C-terminal domains. The C-terminal dimerizing domain of IF_1_ is a single extended α-helix that forms a coiled-coil with another IF_1_. In contrast, the C-terminal α-helix of ζ forms part of a globular four-α-helix bundle that does not seem to promote ζ dimer formation (see Figure 1 and Figure 5). In contrast, the C-terminal domain of ε is the inhibitory domain that forms a two-α-helix hairpin (see Figure 1). This dimerizing C-terminal domain of IF_1_ is, therefore, completely different from the globular and C-terminal domain of ζ. IF_1_ and ζ also have different functional properties; for instance, IF_1_ is well known to be a very good mtF1-ATPase and mtF_1_F_O_-ATPase inhibitor at acidic pH (6.0–6.8) [15], whereas it loses its inhibitory properties at alkaline pH (8.0–9.0). This is consistent with its physiological role, as IF_1_ is supposed to inhibit the mitochondrial F_1_F_O_-ATPase and thus protect the ATP pools when the proton gradient diminishes due to anoxia or ischemia, where the mitochondrial respiratory chain is dysfunctional. Therefore, the proton gradient is partially or totally collapsed, producing a more acidic pH in the mitochondrial matrix. This pH sensitivity is due to a pH sensor of two histidine pairs between the inhibitory N-terminal and dimerizing C-terminal domains of IF_1_; Figure 5 green box and ref [15]. In contrast, the ζ subunit of α-proteobacteria lacks these histidine pairs and therefore works better as an F_1_F_O_-ATPase inhibitor at alkaline, pH = 8.0, than at neutral (pH = 7.0) or acidic (pH = 6.0) pH (see Figure 5) [14]. Thus, IF_1_ and ζ are very different inhibitor proteins that only evolutionarily converged in their N-terminal inhibitory domains and binding sites in their respective F1s (see Figure 1 and Figure 5). However, their origins are different since they emerged separately before and after the mitochondrial endosymbiosis. It could be that the optimal alkaline pH of ζ is necessary for the inhibition of the PdF_1_F_O_-ATPase not only during the collapse of the proton gradient but also in its presence. This is consistent with its inhibitory mechanism since it works as a pawl/ratchet or unidirectional inhibitor, exclusively blocking the CCW rotation of the central rotor in the F_1_F_O_-ATPase direction [41,42,44,50]. Since free-living α-proteobacteria such as *Paracoccus denitrificans* are exposed to more extreme environmental changes than mitochondria, it seems that nature designed a stronger and more efficient PdF_1_F_O_-ATPase inhibitor in free-living α-proteobacteria to prevent any risky ATP deficit from coping with challenging environmental changes in a free-living lifestyle with stronger bacterial stress. The evolutionary rise and loss of the ζ subunit apparently took place before the endosymbiotic event that raised the actual mitochondria, where IF_1_ emerged to inhibit and oligomerize the mtATP synthase that shape the mitochondrial cristae.

## 4. Conclusions

The ε subunit is an essential structural subunit of the F-ATP synthase encoded as such in the ATP operon, connecting F_1_ with F_O_ at the central rotor. Because of this key structural function, its β-barrel connecting F_1_ and F_O_ sectors is strictly preserved. On the other hand, the C-terminal domain of bacterial ε, is partially inhibitory in some bacterial F_1_-ATPases such as those of *E. coli*, *Bacillus PS3*, and so on. However, it is highly variable and therefore non-inhibitory in α-proteobacteria such as *P. denitrificans*. Taking into account the functional, structural, and now phylogenetic evidence, the available data show that ε is a structural but non-inhibitory subunit in α-proteobacteria as it is its homologous δ subunit in mitochondria. This non-inhibitory but structural role of ε was likely transferred from α-proteobacteria to mitochondria during mitochondrial endosymbiosis. Later, the previous α-proteobacterial ε evolved, thus becoming the actual structural δ subunit of the central rotor of the mitochondrial F-ATP synthase. 

In α-proteobacteria, the ζ subunit is essentially an exclusive ORF of this class, and the αPATPsζ gene is located outside both of the ATP operons of α-proteobacterial F_1_ and F_O_ sectors, consistent with the supernumerary and regulatory role of this subunit. Its intrinsically disordered N-terminal side (IDPr) is strictly preserved, whereas its C-terminal α-helical side is much more variable. In contrast to ε, the preserved ζ N-terminus harbors the functional inhibitory domain. On the contrary, in the inhibitory ε subunits, the C-terminal side holds the inhibitory segment. This explains the high conservation of the N-terminal sides of ζ and ε and the highly variable C-termini of both subunits. The primary function of ζ is inhibitory, so the non-inhibitory C-terminal side varies significantly across the α-proteobacterial class. These variations are surely coupled to the changes in the different β and α subunits of F_1_, where the C terminus of ζ forms an anchoring domain interacting with α and β of the F_1_ portion of the α-proteobacterial F-ATP synthases [41,42,44]. 

Although we still may have to show more clearly whether ζ is the evolutionary predecessor of IF_1_ or not, the low identity and similarity between these proteins, the lack of ζ in eukaryotes, and the absence of IF_1_ in procaryotes, particularly in α-proteobacteria, strongly suggest that ζ and IF_1_ (and bacterial ε of course) evolved separately by convergent evolution into the actual inhibitory proteins that prevent wasteful F_1_F_O_-ATPase in α-proteobacteria, mitochondria, and non-α-proteobacteria, respectively. Since the ζ subunit does not seem to be a predecessor of mitochondrial IF_1_ and as ζ is absent in strictly parasitic α-proteobacteria that could be closer to the mitochondrial pre-endosymbionts, we propose that the ζ and ε subunits of the α-proteobacterial F-ATP synthase might be considered from now on as evolutionary tracers to look for the candidates to be closer to the pre-endosymbiont selected by nature to evolve among the α-proteobacteria to become the actual mitochondria. Those α-proteobacteria lacking ζ might be the ones that are more likely to be closer to the mitochondrial pre-endosymbiont, invoked by Lynn Margulis and others [11,12,13,75,76]. This possibility of considering the α-proteobacterial ζ subunit as a mitochondrial pre-endosymbiont tracer gene is reinforced by the loss of inhibitory function of the ε subunit of the F_1_F_O_-ATPase of α-proteobacteria, which eventually became the non-inhibitory mitochondrial δ subunit. Later, when the mitochondrial endosymbiosis was established, the mitochondrial F_1_F_O_-ATP synthases acquired more supernumerary subunits that induce the dimerization and oligomerization of the mtATP synthase which give its shape to the mitochondrial cristae. These mtATP synthase dimers and oligomers are, by the way, also stabilized by the mitochondrial inhibitor protein (IF_1_) [102,108]. Therefore, the adaptative pressure to protect the eukaryotic ATP pools promoted the acquisition of another inhibitor protein after the mitochondrial endosymbiotic event, leading to the emergence of IF_1_, which was acquired with two main functions: to prevent wasteful mitochondrial F_1_F_O_-ATPase activity through its inhibitory N-terminal domain and to stabilize the F-ATP synthase dimers and oligomers by its own dimerization of the C-terminus. This IF_1_ and other F-ATP synthases dimerizing and oligomerizing proteins give their curved shape to the mitochondrial cristae, and so the inner mitochondrial membrane surface available to produce enough ATP is increased to fulfill the large bioenergetic demands of the expensive eukaryotic lifestyle.

## Data Availability

Not applicable.
